# Neoadjuvant radiotherapy in ER^+^, HER2^+^, and triple-negative -specific breast cancer based humanized tumor mice enhances anti-PD-L1 treatment efficacy

**DOI:** 10.3389/fimmu.2024.1355130

**Published:** 2024-04-29

**Authors:** Christina Bruss, Veruschka Albert, Stephan Seitz, Stephanie Blaimer, Kerstin Kellner, Fabian Pohl, Olaf Ortmann, Gero Brockhoff, Anja K. Wege

**Affiliations:** ^1^ Department of Gynecology and Obstetrics, University Medical Center Regensburg, Regensburg, Germany; ^2^ Bavarian Cancer Research Center (BZKF), Regensburg, Germany; ^3^ Department of Radiotherapy, University Medical Center Regensburg, Regensburg, Germany

**Keywords:** in-situ vaccination, breast cancer, neoadjuvant irradiation, checkpoint therapy, combination therapy, humanized tumor mice (HTM)

## Abstract

Pre-operative radiation therapy is not currently integrated into the treatment protocols for breast cancer. However, transforming immunological “cold” breast cancers by neoadjuvant irradiation into their “hot” variants is supposed to elicit an endogenous tumor immune defense and, thus, enhance immunotherapy efficiency. We investigated cellular and immunological effects of sub-lethal, neoadjuvant irradiation of ER pos., HER2 pos., and triple-negative breast cancer subtypes in-vitro and in-vivo in humanized tumor mice (HTM). This mouse model is characterized by a human-like immune system and therefore facilitates detailed analysis of the mechanisms and efficiency of neoadjuvant, irradiation-induced “in-situ vaccination”, especially in the context of concurrently applied checkpoint therapy. Similar to clinical appearances, we observed a gradually increased immunogenicity from the luminal over the HER2-pos. to the triple negative subtype in HTM indicated by an increasing immune cell infiltration into the tumor tissue. Anti-PD-L1 therapy divided the HER2-pos. and triple negative HTM groups into responder and non-responder, while the luminal HTMs were basically irresponsive. Irradiation alone was effective in the HER2-pos. and luminal subtype-specific HTM and was supportive for overcoming irresponsiveness to single anti-PD-L1 treatment. The treatment success correlated with a significantly increased T cell proportion and PD-1 expression in the spleen. In all subtype-specific HTM combination therapy proved most effective in diminishing tumor growth, enhancing the immune response, and converted non-responder into responder during anti-PD-L1 therapy. In HTM, neoadjuvant irradiation reinforced anti-PD-L1 checkpoint treatment of breast cancer in a subtype –specific manner. According to the “bench to bedside” principle, this study offers a vital foundation for clinical translating the use of neoadjuvant irradiation in the context of checkpoint therapy.

## 1 Introduction

Compared to other malignancies such as melanoma or lung cancer, breast cancer (BC) exhibits lower immunogenicity, although this characteristic varies among its individual subtypes. Unlike triple negative (TNBC) and HER2-pos. breast cancer which commonly manifest substantial genomic and phenotypic abnormalities, the estrogen receptor (ER) positive subtype known as luminal, is characterized by lower dedifferentiation and reduced immunogenicity. The extent of immunogenicity correlates with the mutational burden of malignant cells (1, 2) which serves as the basis for the generation of intracellular and cell-surface located neoantigens. The existence, the presentation, and the release of neoantigens by tumor cells may trigger an activation of immune cells, which leads to the development of an immunological anti-tumor defense. This anti-tumor immune response is marked by a pronounced presence of tumor-surrounding (stromal) and infiltrating (intratumoral) lymphocytes. Among a variety of immune cells, cytotoxic T cells are (not the only but) typically the primary actors of tumor cell killing. Due to the varying mutational burden among the BC subtypes, the presence of tumor-infiltrating lymphocytes (TILs) generally tends to increase from the luminal subtype to the HER2-pos. and subsequently to the triple negative subtype (3, 4). A higher abundance of immune cells in HER2-pos (5) and notably in TNBC (6) predicts a more favorable outcome after neoadjuvant chemotherapy. In contrary, in luminal BC TILs are generally less present and elevated TIL rates have been identified as unfavorable prognostic factor and predictor for neoadjuvant treatments (7). Indeed, poor treatment efficiency (e.g., of endocrine treatments) has been reported in several studies in luminal BC exhibiting higher levels of infiltration (8, 9); however, a favorable impact on endocrine treatments has been testified as well (10). Therefore, the relevance of TILs in luminal BC is controversially discussed (11). The attempt to increase the amount of TILs and exploring their therapeutic potential in this subtype is the subject of ongoing preclinical and clinical research.

Nevertheless, elevating the immune cell infiltration and activating the antitumoral immune response is generally considered a promising strategy to reduce the tumor burden and to curb a systemic disease. Neoadjuvant, stereotactic tumor irradiation has the potential to serve as a pivotal component for implementing such a strategy (12). By irradiation of a primary tumor, non-apoptotic cell damage is induced resulting in immunogenic cell death. Thus, initiating the release and presentation of tumor-specific antigens could trigger the activation of the patient’s inherent immune system. More specifically, the recognition of so far concealed neoantigens potentially triggers a patient-individual immunological tumor defense by stimulating T cells, antigen presenting cells, and other immune cells with anti-tumorigenic activity. At best, this activation would also enable an anti-tumor response at distant sites, thereby preventing the formation of metastases. Moreover, the generation of memory cells would empower the immune system to conquer recurrent tumor cells. Consequently, neoadjuvant irradiation ideally equals a highly individual in-situ vaccination. Preclinical evidence already supports the feasibility of this strategy: More than thirty years ago, the involvement of stimulated T cells induced by irradiation was shown to control tumor growth in a mouse model (13). Combining irradiation with the stimulation of the immune response by using Flt3-ligand enhanced tumor antigen presentation. This combination reduced the formation of lung metastases and prolonged survival in two distinct mouse models (14). Moreover, irradiation combined with immune checkpoint inhibition (anti-CTLA-4/PD-L1) resulted in tumor eradication and regression as well as inhibited metastases formation in a murine BC model (15, 16). More recently, studies with T cell receptor (TCR)-transgenic mice have provided clear evidence that irradiating the primary tumor can indeed prime T cells for tumor-associated antigens (17, 18).

Upon activation, immune cells require a counter-mechanism that results in their shutdown preventing an excessive or uncontrolled immune response (19). The expression of checkpoint molecules on immune cells, such as the programmed cell death protein 1 (PD-1) on T cells is known to reflect the anti-tumor reactivity (20) but can lead to the deactivation of the immune cells. The interaction with the corresponding ligand PD-L1 expressed on other cells (or with its soluble form sPD-L1) results in a immunological downregulation (21). The expression of PD-1 on immune cells signifies activation, which in turn results in so-called exhaustion upon interaction with PD-L1. Tumor cells take advantage of this “shut-off” mechanism by expressing PD-L1 (22). Consequently, not only the number of TILs but also the extent of PD-L1/PD-1 interaction mutually determine the anti-tumor activity.

A therapeutic strategy to reactivate immune cells is the application of antibodies targeting these immune checkpoints. Atezolizumab (anti-PD-L1), as well as pembrolizumab and nivolumab (anti-PD-1) demonstrate therapeutic efficiency in treating malignancies characterized by a high mutational burden (23). An anti-PD-(L)1 treatment (in combination with chemotherapy) has been also approved for the treatment of TNBC; however, the success rates are relatively low (24). Multiple studies have demonstrated a lack of a significant benefit for patients, especially in terms of overall survival (OS) (25, 26). However, the predictive value of PD-L1 expression for the response rate to checkpoint therapies, in particular in BC, remains uncertain and is a topic of controversial discussion.

In this study, we evaluated immunologic responses associated with neoadjuvant tumor irradiation leading to reduced tumor growth in BC subtype-specific Humanized Tumor Mice (HTM) in the context of checkpoint immunotherapy. Due to the existence of a human-like immune system, the translational value of this mouse model is exceptionally high, with special regard to studies related to immunotherapies (27, 28). We assessed the treatment efficacy of neoadjuvant tumor specific irradiation in HTMs, of anti-PD-L1 therapy (atezolizumab), or a combination of both. We compared three BC entities with different PD-L1 levels, namely (i) JIMT-1 cells (HER2-pos., trastuzumab resistant, PD-L1-high), (ii) MDA-MB-231 cells (TNBC, PD-L1 moderate), and (iii) MCF-7 cells (ER-pos./HER2-neg., PD-L1 very low). The objective of this study was to identify subtype-specific, treatment-related mechanisms, that allow the optimization and customization of patient-specific, therapeutic interventions by a combinatory approach of neoadjuvant irradiation and immune checkpoint therapy.

## 2 Material and methods

### 2.1 Breast cancer cell lines

JIMT-1 (DSMZ RRID : CVCL_2077), MDA-MB-231 (DSMZ RRID : CVCL_0062) and MCF-7 (DSMZ RRID : CVCL_0031) cells were incubated under standard culture conditions (37°C, 5% CO_2_) in DMEM (MDA-MB-231, MCF-7) or RPMI (JIMT-1) medium supplemented with 5% FCS (Gibco). For experimental approaches, cells were irradiated with 4, 6, 8, or 20 Gy. Irradiations performed for in-vitro analysis were done by using a ^137^cesium source (γ-radiation).

### 2.2 Western blot

Cells were lysed with cell-lysis buffer (Cell Signaling Technology) supplemented with Halt™ Protease (Thermo Fisher Scientific) and phosphatase inhibitor cocktail (Carl Roth). For separation of nuclear and cytoplasmic protein fractions, Nuclear and Cytoplasmic Extraction Reagents was used (Thermo Fisher Scientific). Proteins were separated by 15% SDS-PAGE, were transferred onto polyvinylidene difluoride membranes, were blocked with 5% milk in TBST buffer with 1% Tween for 1 h, and were incubated with primary antibodies in 5% BSA overnight. The following antibodies were used (all from Cell Signaling Technology): H2A.X (RRID : AB_10694556¸1:1000), phospho-H2A.X (RRID : AB_2118010; 1:1000), PD-L1 (RRID : AB_2687655; 1:1000), and Rab11 (RRID : AB_10693925; 1:2000). The detection of actin was realized using the polyclonal rabbit antibody from Sigma-Aldich (RRID : AB_476693; 1:20000). Membranes were incubated with anti-rabbit secondary antibody (AB_2099233; 1:2000) for 1 h at room temperature. Detection was performed by chemoluminescence (SuperSignal west pico PLUS chemiluminescent substrate reagent, Thermo Fisher Scientific) and proteins were visualized using the ChemiDoc Imaging System (Image Lab 6.0.1; BioRad; RRID : SCR_014210).

### 2.3 Immunofluorescence

Cells were seeded in 8-well chamber slides, irradiated with 6 Gy and were fixed with 4% formaldehyde. After blocking with 5% normal goat serum (Cell Signaling Technology), slides were incubated with pH2A.X primary antibody (RRID : AB_2118010) or IgG isotype control antibody (RRID : AB_1550038) overnight at 4°C. Slides were incubated with secondary antibody (goat anti-rabbit IgG AF488, RRID : AB_1904025) for 1 h at room temperature. All antibodies were purchased from Cell Signaling Technology. Slides were covered with Vectashied antifade mounting medium with DAPI (Vector Laboratories). Detection was performed on an inverse fluorescence microscope (AxioImager Z1; Zeiss).

### 2.4 Flow cytometry

Flow cytometric analysis were performed using a BD FACSCanto-II™ flow cytometer (BD Biosciences), which was run by Diva™ software v7.0 (BD Biosciences). Apoptosis was determined for adherent and detached cells 48 or 72 h after irradiation, based on Annexin-V-FITC/DAPI staining.

Cells were stained for ecto-calreticulin (CRT) after 48 h up to 120 h post irradiation, by incubation with CRT primary antibody (FMC 75, Invitrogen) and PE-conjugated secondary antibody (Thermo Fisher Scientific RRID : AB_1954979). DAPI was added 5 min prior flow cytometric analysis.

For cell cycle analysis, cells were fixed and permeabilized in methanol (70%) overnight. The next day, cells were incubated with RNAase (Sigma Aldrich) at 37°C for 20 min, and stained with DAPI (50 µg/ml) for 30 min. DNA histograms were plotted on a linear scale. Cell cycle fractions were quantified by ModFit LT 3.2 software (Verity Software House, RRID : SCR_01610).

For the assessment of polyploidization via Cyclin B1 staining, cells were fixed with 4% formaldehyde, and permeabilized by True-Phos™ Perm Buffer (BioLegend) for 60 min at -20°C. Antibody staining was performed using anti-Cyclin B1-AF647 (BioLegend; RRID : AB_2632638) or the corresponding isotype control. Samples were incubated with RNase (Sigma Aldrich) and DAPI (50 µg/ml), 30 min before measurement.

In order to analyze the possible competitive binding between atezolizumab and the antibody used for flow cytometry, MDA-MB-231 cells were cultivated for 30 minutes in the presence or absence of atezolizumab (10 mg/ml), were stained for PD-L1 and subsequently analyzed by flow cytometry.

Quality control of CD34 isolates was performed using αCD34-Pe (RRID : AB_1731937) and αCD3-FITC (RRID : AB_314060) from BioLegend. The reconstitution of the human immune system in mice was checked in peripheral blood using flow cytometry. To obtain single cell suspension of spleens for the phenotyping of immune and tumor cells, spleens, lungs and tumors were dissociated by passing the cells through a 40 µm cell strainer (BD Bioscience). Bone marrow cells were isolated from the femur flushing the bone cavity with PBS. To reduce nonspecific binding, cells were incubated with 1% mouse serum before staining. The following anti-human antibodies were used and purchased from BioLegend: αCD45-BV510 (HI30, RRID : AB_2561940), αCD8a-BV510 (RPA-T8, RRID : AB_2561942), αNKp46-PeCy7 (9E2, RRID : AB_2561621), αCD33-PerCP-Cy5.5 (WM53, RRID : AB_2074241), αCD45RA-BV421 (HI100, RRID : AB_10965547), αPD-1-AF647 (EH12.2H7, RRID : AB_940471), αPD-L1-BV421 (29E2A3, RRID : AB_2563852), αEpCAM-AF647 (9C4, RRID : AB_756086), αICAM-1-AF647 (HA58, RRID : AB_2715941), αCD45RA-BV421 (HI100, RRID : AB_10965547), αCD56-PeCy7 (51H11, RRID : AB_2563927). αCD3-FITC (UCHT1, RRID : AB_395739), αCD44-BV510 (IM7, RRID : AB_2650923) and αCD4-APC-H7 (SK3, RRID : AB_1645732) and αMHCII-BB700 (Tu39, RRID : AB_2871434) were purchased from BD Biosciences, αCD19-PE (RRID : AB_10734045), αCD24- Pe-Cy7 (eBioSN3, RRID : AB_2573334), αMHCI-PE (MEM-123, RRID : AB_11154825) and αCD27-PECy7 (RRID : AB_1724039) from Thermo Fisher.

### 2.5 Bead-based immunoassay

Soluble molecules in cell culture supernatants and HTM serum were analyzed using the LEGENDplex™ HU Immune Checkpoint Panel 1 (BioLegend Cat# 740867; analyzed molecules: sCD25, 4-1BB, sCD27, B7.2, free active TGFß1, CTLA-4, PD-L1, PD-L2, PD-1, TIM-3, LAG-3, and Galectin-9) and the LEGENDplex™ HU Essential Immune Response Panel (BioLegend, Cat# 740929; analyzed molecules: IL-4, CXCL10, IL-1β, TNF-α, CCL2, IL-6, CXCL8, free active TGF-β1) according to manufacturer’s protocol. Data were processed using the LEGENDplex™ Data Analysis Software Suite.

### 2.6 Generation and treatment of humanized tumor mice

HTM were generated as previously described (29) and illustrated in **Supplementary Figure 1A**. In more detail, human cord blood samples (n=23) were obtained from the Department of Gynecology and Obstetrics (University Medical Center Regensburg) upon signed informed consent and subsequently diluted 1:1 with PBS. Mononuclear cell separation was performed by Pancoll (PAN Biotech) density gradient centrifugation (30 minutes, 600 xg, room temperature) and cells from the interphase were collected and washed twice in EDTA-PBS solution. CD34-pos. cells were isolated using CD34^+^ MicroBeads and FcR Blocking Reagent (Miltenyi Biotec RRID : AB_2848167) according to the manufacturer’s instructions. To increase the purity of HSC, the cells were passed through the LS column twice. Quality control was performed using flow cytometry. NOD.Cg-*Prkdc^scid^ Il2rg^tm1Wjl^*/SzJ (NSG; RRID : IMSR_JAX:005557) mice were housed and bred in a specialized pathogen-free facility at the University of Regensburg. Newborn mice were irradiated (1 Gy) at the age of ~48 hours and mice were intrahepatically transplanted with ~1 × 10^5^ human CD34-pos. cells three hours later. The reconstitution of human immune cells in the peripheral blood was tested eight weeks later by flow cytometry. At the age of nine weeks, humanized mice (when successful engrafted with >10% human CD45-pos. cells in the blood) were transplanted orthotopically in the mammary fad pad with JIMT-1, MDA-MB-231, or MCF-7 tumor cells under anesthesia (midazolam 5 mg/kg, fentanyl 0.05 mg/kg, and medetomidine 0.5 mg/kg i.p.). Anesthesia was antagonized with flumazenil (0.5mg/kg), atipamezol (2.5 mg/kg) and naloxon (1.2 mg/kg). MCF-7 based HTM received 17-beta-estradiol (Sigma-Aldrich^®^) diluted in drinking water (8 μg/ml).

HTM from the same cord blood donor were divided equally in the control and treatment groups. Treatments started when tumors reached 5 mm in diameter. Tumor irradiation with 6 Gy was confined to tumor areal using a linear accelerator (Elekta Synergy S™) and was performed in the Department of Radiation Oncology (University Hospital Regensburg) using an electron linear accelerator. The anti-PD-L1 antibody atezolizumab (5 mg/kg; RRID : AB_2943467) was administered intraperitoneal once a week for a period of five weeks.

### 2.7 Ethic statements

The animal work was approved by the local veterinary authorities of the district government of Bavaria based on the European guidelines and national regulations of the German Animal Protection Act (permission number: 55.2 2-532-2-803). Cord blood samples were taken with approval from the Ethics Committee of the University of Regensburg (permission no. 18-1039-101). All patients provided written informed consent.

### 2.8 Statistical analyses

The results are shown either as median or mean and standard deviation (SD), as described in the figure legends. Statistical analyses were performed using the GraphPad PRISM 6 (RRID : SCR_002798). Data are judged to be statistically significant when *p* ≤ 0.05 according to the one-way ANOVA and Šidák multiple comparison test or Tukey’s multiple comparisons test, two-way ANOVA, and paired parametric t-test, as described in the figure legends. In the figures, asterisks denote statistical significance (* *p* ≤ 0.05, ** *p* ≤ 0.01, *** *p* ≤ 0.001).

## 3 Results

### 3.1 The sensitivity of breast cancer cells to irradiation varies based on their specific subtype

**Figure 1:**
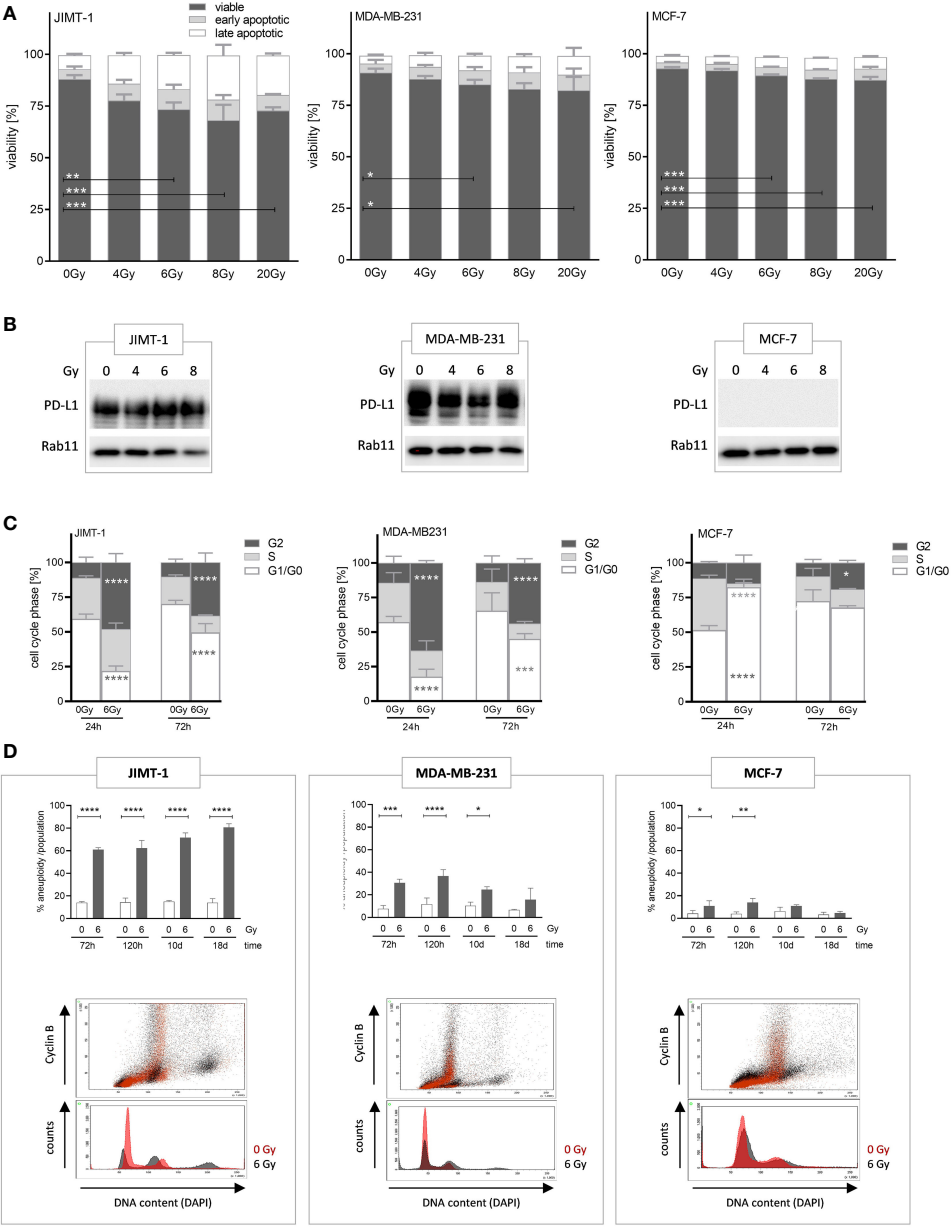
Analyses of irradiation-induced effects on viability, proliferation, and polyploidization in breast cancer cells. **(A)** Apoptotic cell death induced by varying radiation dose was analyzed by flow cytometry. JIMT-1 (HER2), MDA-MB-231 (TNBC), and MCF-7 (HR^+^) were irradiated with 4, 6, 8, or 20 Gy. After 72 h both adherent and detached cells were harvested and stained with Annexin-V and DAPI. The fractions of viable, early, and late apoptotic cells are shown. **(B)** PD-L1 protein expression was determined by western blot after 24 h post annotated irradiation. One representative blot is shown with Rab11 as loading control. **(C)** Cell cycle phases (G0/G1-, S-, and G2/M phase) were quantified by flow cytometry upon DAPI staining 24 and 72 h after irradiation with 6 Gy. Data are given as mean ± SD and two-way ANOVA, Tukey’s multiple comparisons test was applied. **(D)** Irradiation induced polyploidization was quantified based on DNA (DAPI) and Cyclin-B1 staining over a period of 3 to 18 d. The upper panel displays the quantification of unaltered and altered (i.e., polyploidized) cell fractions as a function of 6 Gy irradiation and time, as annotated. Data are given as mean ± SD for JIMT-1 (n=3), MDA-MB-231 (n = 3), and MCF-7 (n = 3) and Šidák multiple comparison test was applied. The lower panel shows an overlaid example measurement 72 h after irradiation. The red-colored histograms represent cells without treatment, while the black colored plots show 6 Gy irradiated cells. Cyclin B1-positive cells with doubled DNA content are considered G2 cells. Cyclin B1-negative cells with doubled DNA represent G1 cells with genome duplication induced upon irradiation. Data are given as mean ± SD; *p ≤ 0.05, **p ≤ 0.01, ***p ≤ 0.001; ****p ≤ 0.0001.

Radiotherapy is a prevalent treatment option for numerous patients with tumors. In the initial phase, we evaluated the radiosensitivity of three distinct breast cancer cell lines, each affiliated with a specific subtype, namely JIMT-1, MDA-MB-231, and MCF-7 cells, and assessed the induction of apoptotic cell death by exposure to different doses of irradiation. The greatest fraction of (early and late) apoptotic cells was found in JIMT-1, followed by MDA-MB-231, and MCF-7 cells, while the number of apoptotic cells increased in a dose-dependent manner (**Figure 1A**). The exposure to 6 Gy induced a moderate level of cell death, but without the eradication of all cells. The sensitivity to 6 Gy irradiation was the lowest in MCF-7 cells with only 10% apoptotic cells, intermediate in MDA-MB-231 cells (19%), and highest in JIMT-1 cells (24%). Based on these data, the dose of 6 Gy was chosen for subsequent in-vitro and in-vivo experiments. In addition, we analyzed whether increasing irradiation doses induce an upregulation of PD-L1 expression, as already shown in the literature. Irradiation did not substantially alter the PD-L1 levels in the analyzed cell lines (**Figure 1B**). However, as described previously (30), the PD-L1 expression is high in JIMT-1 and MDA-MB-231 cells, but absent in MCF-7 cells.

In order to see effects of irradiation on cell proliferation we assessed cell cycle phases, 24 and 72 h after exposure to 6 Gy (**Figure 1C**). We observed a significant delay in cell cycle progress in the G2 phase after 24 h in JIMT-1 (50% vs. 10% arrested in G2 phase: increase 80%) and MDA-MB-231 cells (60% vs. 15%: increase 75%). After 72 h the observed effect was reduced; however, a pronounced delay, that can be considered as a transient cell cycle arrest, was still visible. Conversely, these effects were considerably or nearly absent in MCF-7 cells (12% vs 10% after 24). Instead, upon irradiation MCF-7 cells significantly showed a transient prolongation of the G1-phase (80% in irradiated vs. 50% in untreated cells after 24 h, 38% increase). In addition to these “snapshot assessments”, we observed either a persistent or a transient polyploidization for a period up to 18 d after irradiation depending on the analyzed cell line (**Figure 1D**). Cells with doubled DNA content were considered as cells in the G2 phase when cyclin B1-positive, whereas cyclin B1-negative cells represented cells in the G1 phase with genome duplication induced by irradiation. JIMT-1 cells showed high and further increasing rates of polyploidization within a period of 18 d upon irradiation, whereas the polyploidization in MDA-MB-231 was visible early after irradiation but was no longer detectable 18 days post irradiation. Irradiation-induced polyploidization in MCF-7 cells was just marginal and not traceable from day 10 post-irradiation (**Figure 1D**).

### 3.2 Pronounced DNA damage and immunogenicity in JIMT-1 and MDA-MB-231 cells upon 6 Gy irradiation in-vitro as determined by H2A.X phosphorylation, calreticulin presentation, and cytokine release

**Figure 2:**
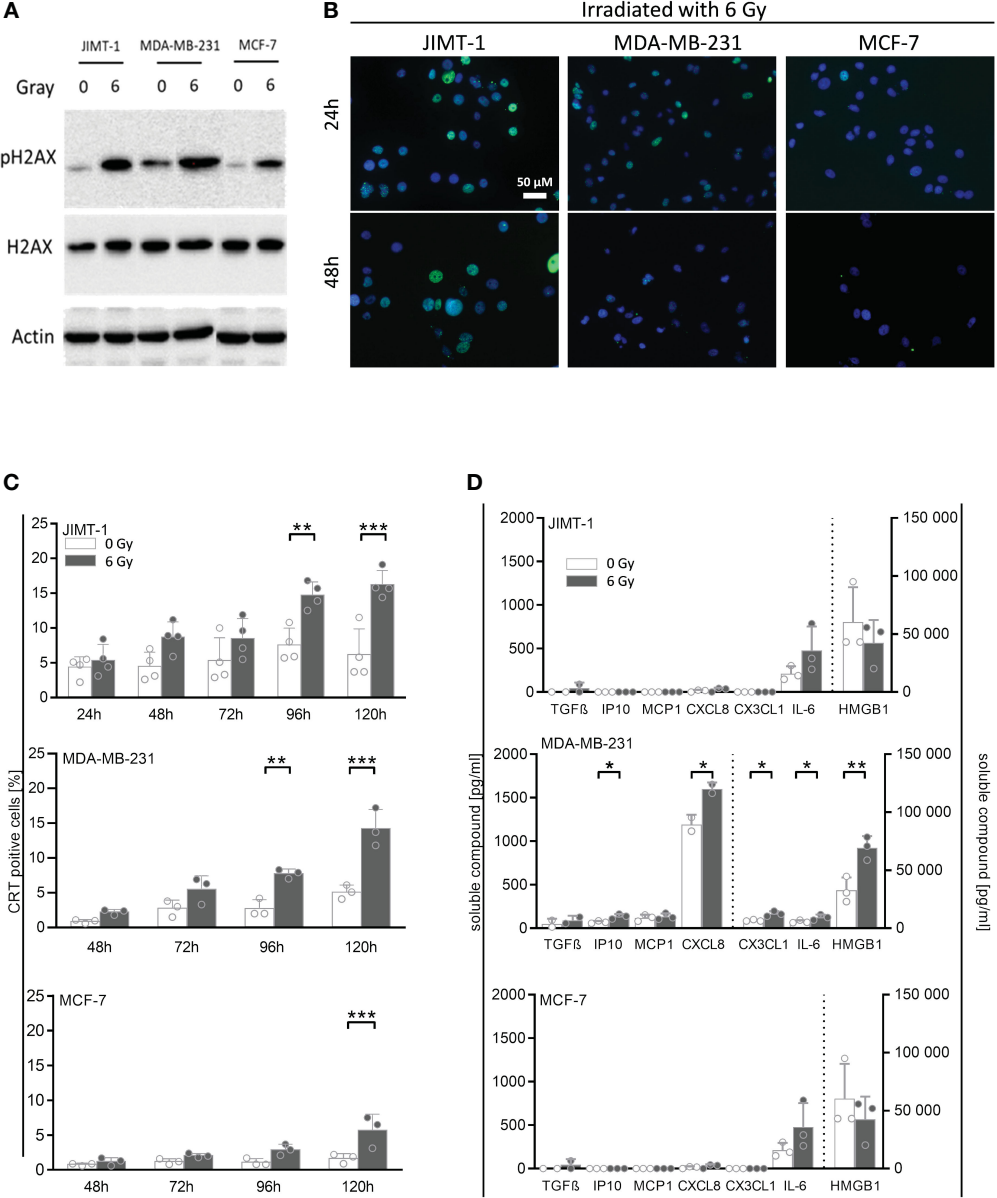
Immunogenic cell death and release of DAMPs and other cytokines induced by irradiation in breast cancer cells. **(A)** Exemplary Western Blot of pH2A.X and total H2A.X after 30 minutes post 6 Gy irradiation. **(B)** Double-strand DNA breaks were analyzed by immunofluorescence staining of pH2A.X (green fluorescence) 24 h and 48 h post irradiation in breast cancer cell lines: JIMT-1 (HER2), MDA-MB-231 (TNBC), and MCF-7 (HR^+^). Nuclei were stained with DAPI (blue fluorescence). **(C)** Calreticulin surface expression was measured by flow cytometry at indicated time points post irradiation in-vitro. One-way ANOVA, Šidák multiple comparisons test was applied, with n = 4 for JIMT-1, n = 3 for MDA-MB-231 and MCF-7, **p ≤ 0.01 and ***p ≤ 0.001. **(D)** Cytokines and other soluble factors associated with immunogenicity were analyzed by flow cytometry and ELISA. Up front, cells were cultured for 72 h under normal cell culture conditions (5% FCS). After medium exchange, the cells were irradiated (6 Gy) and cultured for additional 48 h under starving conditions (1% FCS). Supernatants were collected and HMGB1 and CX3CL1 (fractalkine) secretion was analyzed via ELISA. The human TGF-ß, CXCL10 (IP-10), MCP-1 (CCL2), CXCL8 (IL-8), and IL-6 concentrations were determined via bead-based immunoassays using flow cytometry. The mean ± SD in pg/ml is shown, n = 3. Dashed lines indicate the belonging of data to the right or left y-axes. Conditions were compared via paired t-test, *p ≤ 0.05, **p ≤ 0.01.

In order to analyze whether irradiation can affect the immunogenicity of tumor cells, we assessed a number of cell-associated and soluble markers for immunogenicity and immunogenic cell death, respectively (**Figure 2**). In association with irradiation-induced polyploidization we observed the generation of double-strand DNA breaks by immunofluorescence staining of pH2A.X. About half of the JIMT-1 cell nuclei were seen positive for pH2A.X after irradiation (**Figure 2B**). Roughly 10% of the MDA-MB-231 cells were pH2A.X positive 24 h after irradiation; however, 24 h later, pH2A.X was nearly undetectable. In irradiated MCF-7 cells, there was no evidence for H2A.X phosphorylation. Verifying these results obtained by microscopy, the amount of pH2A.X analyzed by western blot, was also high in JIMT-1, followed by MDA-MB-231, and MCF-7 cells in comparison to control cells (**Figure 2A**). Overall, the cell-specific amount of DNA damages represented by H2A.X phosphorylation correlated with the degree of preceding polyploidization (**Figure 1D**). Moreover, we quantified the cell surface located Calreticulin (CRT) as a marker for immunogenicity (**Figure 2C**). We identified a significant irradiation-induced change of CRT expression after 96 h and 120 h respectively in JIMT-1 and MDA-MB-231 cells. Nevertheless, in MCF-7 cells, the total CRT levels were comparatively rather low. The release of growth factors and cytokines may also be indicative for immunogenicity. TGF-β, IP10, MCP1, CXCL8, CX3CL1, IL-6, and HMBG1, were analyzed, as they were already shown to be secreted by BC cells (**Figure 2D**). Compared to control cells, a significant increase of IP10, CXCL8, CX3CL1, IL-6, and HMBG1 secretion was only seen in MDA-MB-231 cells. Irradiation in JIMT-1 and MCF-1 did not change the profile of secreted factors. Taken together, pronounced secretion and irradiation-induced immunogenic alterations were found in MDA-MB-231 cells, whereas JIMT-1 and MCF-7 cells were only marginally affected.

### 3.3 Neoadjuvant irradiation and concurrent anti-PD-L1 treatment is efficient to curb tumor growth and to stimulate an immune defense in HTM

**Figure 3:**
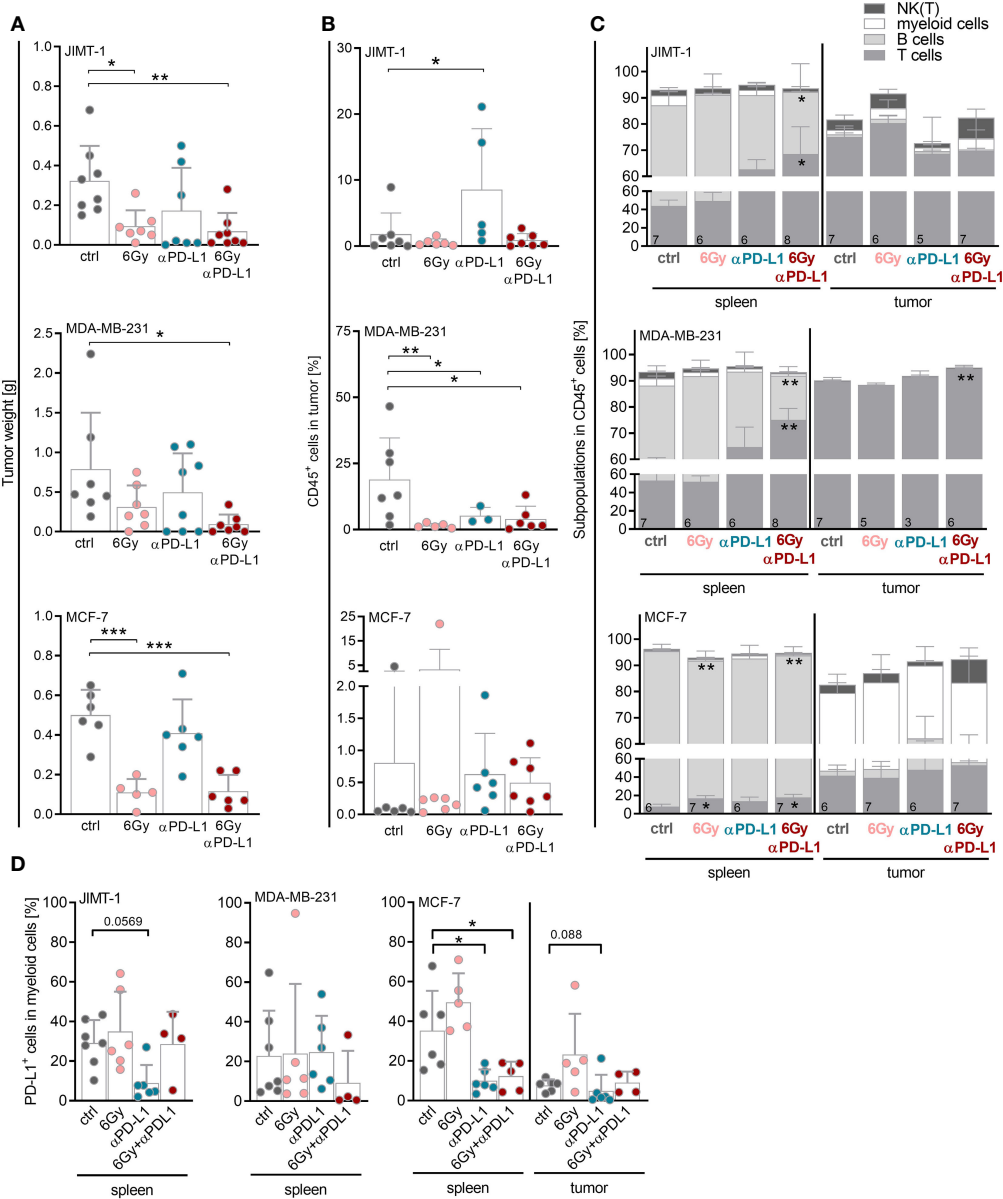
Neoadjuvant irradiation augments the efficacy of checkpoint therapy in HTM with JIMT-1 and MDA-MB-231 tumors possibly by systemic upregulation of T cell abundance. JIMT-1, MDA-MB-231, and MCF-7 breast cancer cells were transplanted orthotopically into humanized NSG mice. Therapy was started when tumors were palpable (about 50 mm^3^). In the case of irradiation, only tumor areal was irradiated with 6 Gy. Anti-PD-L1 antibody (5 mg/kg body weight) was administered i.p. weekly, for treatment regimen see also **Supplementary Figure 1A**. **(A)** Tumor weight was measured for evaluation of response rate to therapy at the end of the experiment. **(B, C)** The tumors and spleens were processed to a single-cell suspension and the cells were subsequently analyzed by flow cytometry. **(B)** Percentage of CD45^+^ cells infiltrated into tumors is shown (median, each symbol represents one individual tumor). **(C)** Composition of immune cell populations among CD45^+^ cells was analyzed by CD3, CD19, CD33 and CD56/Nkp46 staining in spleens and tumors. **(D)** PD-L1 surface expression was analyzed by PD-L1 staining on CD33^+^ cells in spleens and tumors. Due to too little events of CD33^+^ cells in the tumor of JIMT-1 and MDA-MB-231 mice, only spleen is depicted. **(A, B, D)** One-way ANOVA, Tukey’s multiple comparisons test was applied, *p ≤ 0.05, **p ≤ 0.01 and ***p ≤ 0.001. The mean ± SD is depicted, each symbol represents an individual tumor under indicated conditions. **(C)** Data are shown as mean ± SD, n is given in the lower left of each bar and two-way ANOVA, Tukey’s multiple comparisons test was applied, *p ≤ 0.05, **p ≤ 0.01.

In order to analyze the potential of neoadjuvant irradiation in the context of immune checkpoint blockade, JIMT-1, MDA-MB-231 or MCF-7 tumor cells were inoculated into the mammary fat pad of humanized mice. Mice were irradiated with 6 Gy, treated with anti-PD-L1 or a combination thereof (**Supplementary Figure 1A**). A single 6 Gy irradiation of the tumor areal was sufficient to prevent tumor growth in JIMT-1 and MCF-7, but not in MDA-MB-231 transplanted HTM (**Figure 3A**; **Supplementary Figure 1B**). MCF-7, which do not express PD-L1, did not respond to single checkpoint blockade. Similar to the in-vitro experiments (**Figure 1B**), the levels of PD-L1 (as well as MHC I, MHC II, CD44 or CD24; **Supplementary Figure 2A**) remained unchanged upon irradiation in the HTM model analyzed. The putative significance of PD-L1 expression in MDA-MB-231 HTM treated with anti-PD-L1 antibody mice is probably due to a competitive binding of atezulizumab and the diagnostic antibody in case mice were sacrificed near-time of the last injection (**Supplementary Figure 2B**). Among JIMT-1 and MDA-MB-231 mice, the anti-PD-L1 group was found divided into responder and non-responder. The combination of irradiation and anti-PD-L1 therapy was most effective, and resulted in a significantly reduced tumor growth in all three mouse models (**Figure 3A**). These effects seem not to be related to increased numbers of immune cells in the tumor, as infiltration rates were not significantly affected or even lower (MDA-MB-231) compared to the control group (**Figure 3B**). However, MDA-MB-231, mimicking TNBC, were characterized by the highest amount of infiltrated CD45-pos. immune cells in the tumor, in particular when compared to the luminal MCF-7 control HTM. Notably, there was a significant increase in immune cell infiltration in JIMT-1 HTM treated with anti-PD-L1 (**Figure 3B**); however no alterations were noted in HTMs subjected to combination therapy. It is important to note, that in certain mice, therapy was highly effective, resulting in the complete removal of tumors. Consequently, the composition of infiltrating immune cells could not be analyzed.

In order to assess the treatment’s impact on metastatic potential, the presence of tumor cells in the lung or in the bone marrow was analyzed by flow cytometry. There were no substantial differences in the percentage of disseminated tumor cells in the lung (**Supplementary Figure 3A**) or in the bone marrow (**Supplementary Figure 3B**). However, there was a trend towards reduced tumor cells in the lung upon irradiation in JIMT-1 HTM. Due to the experimental constraints, notably the restricted time frame, it was not feasible to clarify whether irradiation could contribute to the abscopal effect. Overall, HTM transplanted with MDA-MB-231 exhibited the highest numbers of disseminated tumor cells in both organs. In order to assess the impact of neoadjuvant irradiation on the immune response, the immune cell composition was characterized in the spleen, reflecting systemic effects, and in the tumor.

Monitoring weight from the start to the completion of therapy revealed an increase in weight for the control groups in JIMT-1 and MDA-MB-231 HTM. Throughout the treatment, the weight gain was mitigated, mostly in the JIMT-1 HTM group receiving combined therapy (**Supplementary Figure 3C**). A shorter time period before the initiation of therapy in MCF-7 HTM, resulted in a reduced amount of weight gain. Similar to the observations in the JIMT-1 and MDA-MB-231 HTMs, MCF-7 HTMs treated with 6 Gy irradiation with or without checkpoint therapy exhibited a negative impact on weight development.

Notably, the immune cells in the spleen varied between the mice bearing JIMT-1 or MDA-MB-231 tumors, displaying more immunogenic models, from the MCF-7 mice. However, it is essential to emphasize that due to the experimental setup, MCF-7 mice were approximately four weeks younger when the therapy was initiated. In JIMT-1 and MDA-MB-231 mice, the predominant splenic immune cell population consisted of T cells, whereas in MCF-7 mice the B cell compartment was more prominent (**Figure 3C**). Strikingly, the T cell numbers significantly increased in the spleen in all three models after combinatory therapy.

Irrespective of the treatment, T cells were the predominant immune cell population in the tumor in JIMT-1 and MDA-MB-231 mice, whereas CD33^+^ myeloid cells constituted the most prevalent population in MCF-7 tumors (**Figure 3C**). Myeloid cells found in the spleen from MCF-7 mice exhibited a significantly reduced percentage of PD-L1^+^ cells after anti-PD-L1 treatment, regardless whether it was combined with irradiation. A similar pattern was observed in the tumors of MCF-7 HTM, as well as for myeloid cells in JIMT-1 mice treated with anti-PD-L1 alone; however, this trend was not evident in the MDA-MB-231 model (**Figure 3C**). Due to the limited presence of intratumoral CD33^+^ cells in JIMT-1 and MDA-MB-231 mice, the analysis of PD-L1 levels was not feasible.

**Figure 4:**
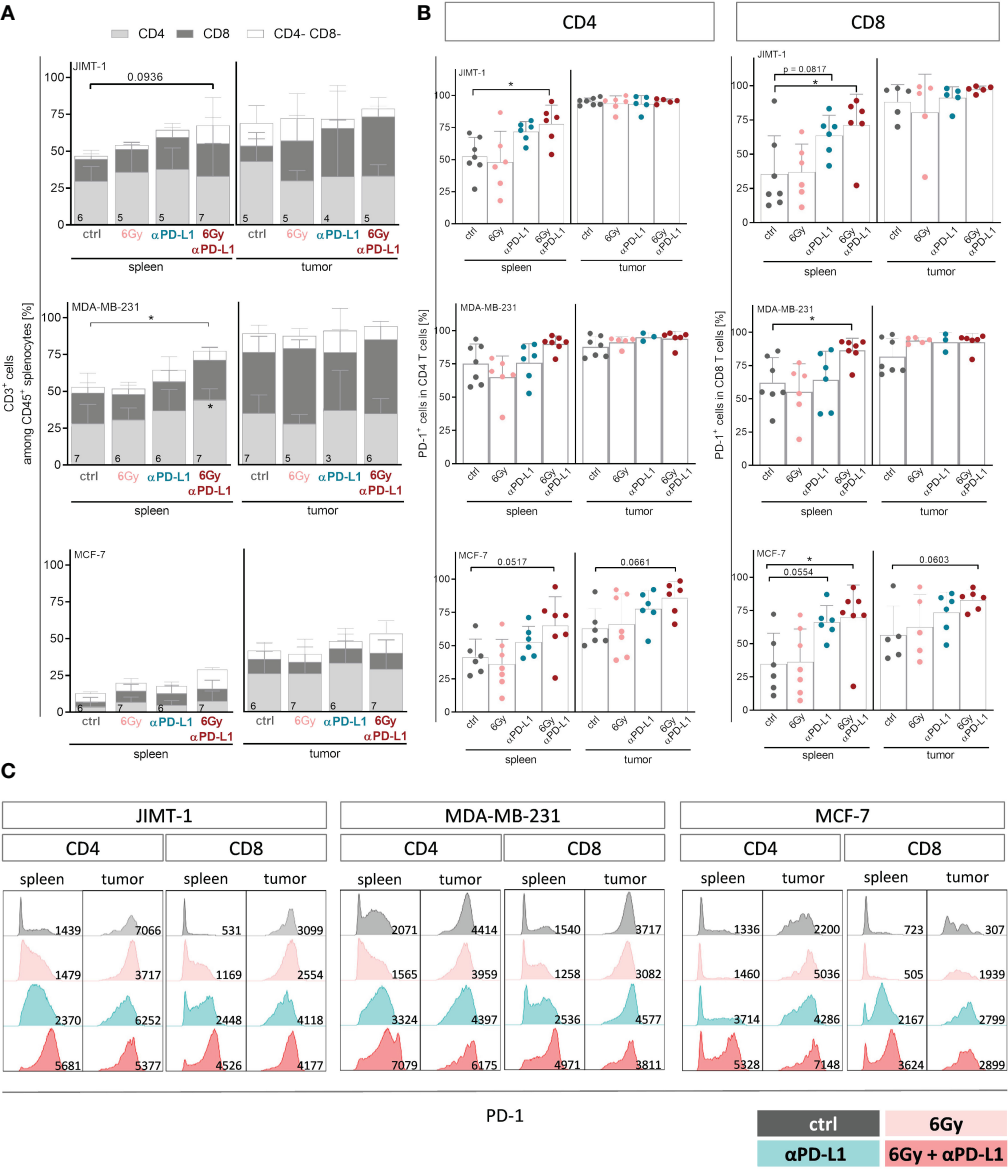
Neoadjuvant irradiation with subsequent checkpoint therapy systemically induces activation of CD4 and CD8 T cells in HTMs. For treatment regimen see **Supplementary Figure 1**. **(A)** T cell amount among CD45^+^ cells and composition thereof were analyzed by flow cytometry via CD3, CD4 and CD8 staining and **(B, C)** CD4 as well as CD8 isolated from spleens and tumors were characterized regarding their PD-1 expression. **(A)** Data are shown as mean ± SD, n is given in the lower left of each bar. **(B)** PD-1 expression is shown as mean ± SD while each symbol represents an individual spleen or tumor. **(A, B)** One-way ANOVA, Tukey’s multiple comparisons test was applied, *p ≤ 0.05. **(C)** Representative FACS blots show PD-1 expression under indicated conditions for CD4 and CD8 T cells isolated from spleen and tumor, MFI is given in the lower right part of each blot.

For a more detailed characterization of T cells, we examined the CD4 and CD8 distribution among CD3^+^ cells (**Figure 4A**), their PD-1 status (**Figures 4B, C**) and their subset composition both in spleens and tumors (**Supplementary Figure 4**). While in spleens of MCF-7 mice the overall low levels of CD4 and CD8 remained unchanged, the predominant CD4 T cell population in the spleen of MDA-MB-231 HTM significantly increased with combination therapy (**Figure 4A**). Interestingly, the prevalent population in the tumors of both MDA-MB-231 and JIMT-1 belonged to the CD8 subset. In order to delineate the subset composition of CD4 and CD8 T cells, CD27 and CD45Ra were used to distinguish between naïve (NV, CD27^+^ CD45Ra^+^), central (CM, CD27^+^ CD45Ra^-^) and effector memory (EM, CD27^-^ CD45Ra^-^), as well as exhausted T cells that re-express CD45RA (CD27^-^ CD45Ra^+^) (**Supplementary Figure 4**). In the spleens of JIMT-1 and MDA-MB-231 HTMs, the CD4 T cell compartment predominantly consisted of more experienced central and effector memory T cells, whereas substantial presence of naïve cells was found in spleens of MCF-7 mice (**Supplementary Figure 4A**). However, the memory CD4 T cells seemed to be systematically increased after irradiation and PD-L1 blockade in all three models. Generally, CD4 T cells found in the tumor belonged to the more experienced subsets, while naïve cells were less frequent.

Elevated levels of naïve CD8 T cells were found in the spleens of all three HTM models (**Supplementary Figure 4B**). Checkpoint therapy alone or in combination with neoadjuvant irradiation contributed to increased frequencies of central memory cells in JIMT-1 and MCF-7, but not in MDA-MB-231 mice. Unlike the CD4 T cell subset distribution, which exhibited a similar trend in all three models analyzed, the composition of CD8 TILs differed among the HTM models. In JIMT-1 mice, intratumoral T cells were predominantly assigned to the more experienced central and effector memory subsets, with the effector memory compartment being particularly pronounced. In MDA-MB-231 mice, a substantial number of cells were identified as central memory T cells, with the additional presence of naïve T cells. The strongest effect was seen in tumors of MCF-7 mice, showing elevated frequencies of exhausted T cells, particularly in mice exposed to irradiation. Notably, the exhausted phenotype could be reversed through the application of checkpoint therapy, ultimately returning to control levels.

While splenic CD4 and CD8 T cells exhibited moderate levels of PD-1 in untreated mice, CD8 T cells became highly activated after combination therapy in all three models (**Figure 4B**). A trend to increased PD-1 expression was also detected on TILs in MCF-7 HTM, whereas PD-1 expression in MDA-MB-231 and JIMT-1 HTM reached already nearly 100% (**Figure 4B**). Interestingly, the expression and intensity of PD-1 varied among mouse models and treatment groups (**Figure 4C**). PD-1 intensity was upregulated especially on CD8 T cells after anti-PD-L1 application with or without irradiation. Moreover, the serum of HTMs was examined for the soluble form of PD-1 (sPD-1) (**Supplementary Figure 5A**). The levels of sPD-1 were relatively low in all three HTM models, with MCF-7 control mice exhibiting the lowest levels compared to JIMT-1 and MDA-MB-231 mice. Exposure to irradiation alone seemed to induce sPD-1 secretion in MCF-7 mice, although this effect was not observed in combination with anti-PD-L1 treatment. Interestingly, elevated levels of several compounds including sCD25, sCD27, 4-1BB, B7.2, sPD-L2, galectin-9 and sLAG-3 were detected not only in the irradiation group, but also in other groups, especially those undergoing combination therapy (**Supplementary Figure 5B**). In order to investigate the potential of soluble checkpoint molecules as indicators of treatment response to checkpoint blockade, we analyzed levels of soluble compounds in MDA-MB-231 mice categorized as responder and non-responder after single anti-PD-L1 (**Supplementary Figure 5C**). Given the limited numbers of analyzed mice, the results were not definitive, but there was a trend for several soluble compounds, that were different in responder mice in comparison to control and non-responder mice. These compounds included sCD27, sPD-1, sPD-L1, sPD-L2, sTIM-3, and others.

## 4 Discussion

In this study, we evaluated irradiation-induced cellular effects and the irradiation sensitivity of three subtype-specific BC cell lines, namely JIMT-1 (HER2-pos., trastuzumab resistant), MDA-MB-231 (triple negative), and MCF-7 (ER-pos., i.e., luminal) in-vitro. Moreover, we assessed efficacies of neoadjuvant irradiation in HTM models with a human-like immune system. We identified potential alterations specific to immune cells and examined the immunological anti-tumor defense in response to irradiation, anti-PD-L1 treatment alone or in combination.

Before starting treatment experiments with HTM we evaluated cellular and molecular effects induced by different irradiation doses (up to 20 Gy) in-vitro. Read out parameters were the generation of pH2AX, the inhibition of cell proliferation and potential cell cycle arrest, the generation of enhanced polyploidy, the cellular presentation of CRT and last but not least the fraction of apoptotic cells. Aim was to elicit just moderate alterations without inducing significant amounts of cell death assuming that irradiated but in particular still vital cells have the capacity to induce an immunological anti-tumor effect. We found a single irradiation dose of 6 Gy most appropriate to induce detectable events without considerable amounts of apoptosis. In contrast, lower doses were inefficient to elicit detectable alterations, while the application of higher (and or repeated) resulted in inacceptable amounts of cell death.

More precisely, we monitored the irradiation-induced generation of pH2AX as maker for DNA damages over a short (**Figure 2A**) and a long (**Figure 2B**) period of time upon irradiation and visualized the results either by Western Blotting (short time) or immunofluorescence (long time). Phosphorylation of H2A.X is known as a very early event in response to DNA double-strand breaks (31) and represents a prerequisite for the recruitment of DNA repair factors. However, H2AX becomes dephosphorylated during DNA repair (32), which indicates successful DNA repair and survivability of (in this case irradiated) cells.

Covering short and long term monitoring revealed that despite high pH2AX levels in MDA-MB-231 shortly after irradiation-induced DNA damage, these cells virtually do not show any visualizable pH2AX after 48 h. In contrast, detectable phosphorylation still persists after 48 h post irradiation in JIMT-1 cells which correlates with the generation of higher rates of apoptotic cells (**Figure 1A**) and an increased degree of aneuploidy later on (**Figure 1D**). Taken together, these events unambiguously indicate highest sensitivity to irradiation of JIMT-1 cells, moderate sensitivity in MDA-MB-231 cells, and lowest sensitivity of MCF-7 cells. MCF-7 cells, show lowest pH2AX levels at early and late time points (**Figure 2A**) that indicates an efficient DNA repair activity in this cell line. Comparing alterations in all three cell lines the responsiveness to irradiation (in terms of DNA damages indicated by pH2AX) was most pronounced in JIMT-1 and gradually decreases over MDA-MB-231 to MCF-7 cells in a dose-dependent manner.

Alongside the objective to preserve manipulated by mainly vital cells single dose irradiation instead of fractionated scheme was later on applied for in-vivo studies in order to avoid inhibitory effects of irradiation on infiltrating lymphocytes. Irradiation induced negative effects have been reported, for example, by a study in which T cell propagation and activity is reduced upon irradiation with 4 Gy (or higher doses) (33).

Irradiation doses up to 20 Gy were generally sublethal, even though JIMT-1 cells exhibited 20 - 25% apoptotic cells, whereas MCF-7 cells were mainly irresponsive and remained predominantly viable. The distinct cell-inherent radio resistance observed in MCF-7 cells may, to so some extent, be attributed to the ability of luminal BC cells to undergo phenotypic changes and exhibit cellular plasticity, as demonstrated by Gray et al. (34). Additionally, the same group reported modifications in intracellular signaling, revealing a diminished estrogen receptor presence but simultaneously an increased MAPK activity upon exposure to irradiation. Nevertheless, the physical generation of DNA damages by identical dosages applied to different cell types is supposed to be equivalent. It appears plausible that the capacities to manage potential genome damage vary among distinct cell types. The molecular proficiency for DNA repair obviously decreases from MCF-7 to MDA-MB-231 and further to JIMT-1 cells. This notion seems plausible, given that a higher mutational burden has consistently been attributed to HER2-pos. and triple negative BCs in comparison to the luminal subtypes. A defective DNA repair mechanism can entail the elimination of cells containing severe DNA damage through apoptotic cell death or may contribute to the propagation of daughter cells with further accumulated DNA damage and modifications. The latter phenomenon may in particular pertain to JIMT-1 cells, characterized by an inherent (hyper-)active PI3K, a factor driving cell survival (35). The constitutive PI3K activity might ensure the survival of JIMT-1 cells despite damaged DNA, potentially explaining the manifestation of irradiation-induced polyploid JIMT-1 cells (see below).

In our previous research, we have shown that in particular JIMT-1 but also MDA-MB-231 cells express PD-L1 (30). In contrast, PD-L1 was scarcely detectable in MCF-7 cells. The intrinsic PD-L1 expression in surviving cells remained unaffected by irradiation across all cell lines (**Figure 1B**). This observation contradicts findings from other preclinical research, such as those conducted by Wang (36) or Deng and colleagues (16), who reported an increased PD-L1 expression upon irradiation in mouse mammary tumor models (BALB/c, C57BL/6). However, our data align with observations made in clinical settings (37).

Irradiation triggered a profound G2 cell cycle arrest in JIMT-1 and MDA-MB-231, underscoring the pronounced sensitivity of these cell types to irradiation (**Figure 1C**). Mechanistically, irradiation primarily affects proliferating cells and causes DNA damage principally during the S-phase. Accordingly, an arrest in the G2-phase should facilitate the repair of potential DNA damage. If repair mechanisms are not feasible, cells undergo cell death. Indeed, a notable number of cells (approximately 10% of MDA-MB-231 and 20% of JIMT-1 cells) did not survive the preceding irradiation, despite the application of a moderate dose (6 Gy). Genome modifications, nonetheless, manifest to some extent in surviving cells, as confirmed by pronounced and moderate polyploidization observed in JIMT-1 and MDA-MB-231 cells after irradiation (**Figure 1D**). The degree of irradiation-induced polyploidization in MDA-MB-231 and JIMT-1 cells correlated with the extent of the preceding G2 cell cycle arrest. This arrest facilitates the generation of daughter cells with a multiplied genome e.g., achieved through a process called endoreplication (38). In contrast, MCF-7 cells did not arrest in the G2 phase and did not develop cells with an increased DNA content. This aligns with the virtual absence of the cellular response in-vitro. We substantiated the varying degrees of sensitivity through the identification of irradiation-induced elevated levels of pH2A.X in JIMT-1, moderate levels in MDA-MB-231, but the absence of H2A.X phosphorylation in MCF-7 cells (**Figures 2A, B**). The generation of pH2A.X preceded and instigated the G2-arrest, while the cell surface localized CRT is part of the induced cellular immunogenicity (**Figure 2C**). CRT-pos. cells underwent (irradiation-induced) DNA and cell damage and necessitated an immunological elimination (39). Supposedly, the increased immunogenicity enables and entails immunogenic cell death, it promotes the uptake of cell components by mono- and phagocytes, and ultimately supports the initiation of anti-cancer immunity. In accordance with cell viability and proliferation, the fraction of irradiation-induced CRT-pos. cells was highest in JIMT-1, moderate in MDA-MB-231, and nearly absent in MCF-7 cells.

We assumed that the induction of cellular immunogenicity and tumor-associated immunity is concomitant with the release of a variety of soluble compounds, interleukins and cytokines. A basal HMGB1 release was observed in all three cell types, but the levels increased significantly upon irradiation only in triple negative MDA-MB-231 cells (**Figure 2D**). Notably, only MDA-MB-231 cells were characterized by the release of additional immune stimulating factors, namely IP10, CXCL8, CXCL3, and IL-6, which were significantly enhanced upon irradiation. The corresponding HTM showed the strongest immune cell infiltration in the tumor (**Figure 3B**) but accompanied by enhanced metastases formation (**Supplementary Figures 3A, B**). All of these molecules may have counteractive functions; for instance, HMGB1 belongs to the danger associated molecules (40) and contributes to the activation of the innate immune system, yet it is also documented to enhance the migration and invasion of MDA-MB-231 cells (41). CXCL8 serves as a crucial chemokine for attracting neutrophils during infection. But its angiogenic and inflammatory effects also trigger uncontrolled tumor growth and the formation of metastases (42). CX3CL1 is another important chemoattractant recruiting immune cells toward tumor tissue and has been found to be a positive prognostic factor in numerous cancers, including BC (43). However, if the receptor is present on tumor cells, CX3CL1 has been described to exhibit pro-tumorigenic and pro-metastatic properties. IL-6, an important regulator of inflammation, activates especially the B cell response. However, it also promotes tumorigenesis by regulating multiple cellular processes, including proliferation, angiogenesis, invasiveness and metastasis (44).

The number of infiltrating immune cells in MDA-MB-231 HTM was significantly diminished across all treatment groups (**Figure 3B**). The unexpected reduction of TILs upon irradiation across all HTMs, aligns with the situation observed in advanced BC patients undergoing radiotherapy (mainly HR-pos. and HER2-neg., or TNBC) (37). The timing of sample collection (the duration elapsed after treatment) could be of potential relevance, given that samples were not obtained immediately after treatment (similar to the conditions in this study).

Irradiation alone was proved effective in reducing tumor growth and had a significant effect in both the HER2-pos. and the luminal HTM models (**Figure 3A**). Considering the marked resistance of MCF-7 cells to irradiation in-vitro, the significant decrease in tumor mass observed in irradiated MCF-7 HTM was quite unexpected. Possibly, multifactorial mechanisms could contribute to the in-vivo situation, such as irradiation-induced reactive oxygen species, which may ultimately lead to cell death (45), as well as more prolonged processes such as senescence - a phenomenon that could potentially end up in cell death at a later stage. Indeed, the latter phenomenon has been reported for MCF-7 (46).

Strikingly, in MDA-MB-231 (with an intermediate PD-L1 expression) and JIMT-1 (with a high PD-L1 expression) HTM, responder and non-responder mice were observed when subjected to anti-PD-L1 treatment alone (**Figure 3A**). Conclusively, neither a pronounced PD-L1 expression nor an elevated immune cell infiltration guarantee a sufficient anti-PD-L1 treatment efficiency. This observation reflects the clinical scenario accurately, as the PD-L1 status does not reliably predict response or resistance to anti-PD-L1 treatment (47). Accordingly, reports exist indicating both a positive (48) and a negative correlation (49) between PD-L1 expression and therapy response. Remarkably, the PD-L1 expression on TILs seem to hold a higher predictive value for anti-PD-L1 treatment in TNBC patients compared to the expression on tumor cells (50, 51). This might be attributed to a distinct regulation of PD-L1 by environmental factors, such as IFNγ, which has been described to induce PD-L1 expression on tumor cells (52). However, besides the effect by a competitive binding of atezolizumab and the diagnostic antibody in HTM, which were sacrificed near-time of the last injection, the membrane-bound PD-L1 expression remained unchanged through treatment in all HTM models.

To overcome non-responsiveness, there is a necessity for combined treatment approaches to amplify the effectiveness of single checkpoint therapies (53). This represents the most important and potentially clinically relevant finding of this study. More precisely, we observed a pronounced and significant treatment efficiency in all three BC subtype HTM when subjected to combined irradiation and anti-PD-L1 treatment (**Figure 3A**). The suppression of tumor growth attained through the combined treatment was associated with an increased systemic proportion of T cells and increased PD-1 expression on CD8 T cells in the spleen. Notably, these data align with clinical observations, demonstrating an increased PD-1 expression on infiltrating immune cells, in particular in early TNBC (54) and untreated HR-pos. BCs (55, 56). In addition, we observed an increased proportion of CD4 effector cells in the spleen of JIMT-1 and MDA-MB-231 mice, as well as in the tumor of JIMT-1 HTM upon irradiation combined with anti-PD-L1 treatment (**Supplementary Figure 4**). The activation of CD4 T cells additionally indicated a treatment-induced immune cell stimulation.

Notably, the efficacy of an anti-PD-L1 treatment alone was limited in MCF-7 HTMs but could be enhanced when combined with radiotherapy. We found evidence for an immune cell activation arising from a blockade of the interaction with PD-L1 on immunosuppressive cells since a combined treatment approach triggered PD-1 expression and T cell maturation in all three HTM models. Strikingly, the PD-L1 expression on myeloid cells was significantly reduced upon combined treatment in MCF-7 HTM (**Figure 3D**). However, the distinction of reduced expression or impeded detection because of atezolizumab binding is not possible. The immune cell activation in MCF-7 HTMs is evident through the increased PD-1 expression on tumor infiltrating T cells. The combination therapy brought the PD-1 levels close to those found in MDA-MB-231 and JIMT-1 HTMs. Thus, neoadjuvant irradiation might indeed transform an immunological “cold” tumor into its “hot” counterpart by activating cytotoxic cells and reducing the immune suppression.

Besides the cell-associated immune checkpoint molecules their soluble variants may also influence tumor growth, metastasis formation and response to therapy. Therefore, we evaluated soluble checkpoint molecules in the serum of HTM (**Supplementary Figure 5**). Nevertheless, Rapoport and colleagues did not observe a relation of soluble checkpoints or cytokines with pathological complete response in early BC patients, potentially due to the diversity of the mixed cohort (57). Others described sPD-L1 as a favorable predictive tumor marker in advanced BC patients receiving checkpoint therapy (58). Interestingly, s4.1BB and sPD-1 were significantly increased in irradiated MCF-7 HTMs, which potentially indicates enhanced immune cell activation. Nevertheless, additional studies specific to BC subtypes are essential to comprehensively assess the impact and exploitation of these soluble molecules.

The effect of local irradiation on the formation of metastases is still a subject of ongoing debate and disagreement. In the context of irradiation, there are circulating preclinical and clinical reports discussing increased metastasis formation and cases of tumor regression accompanied by reduced metastases, probably attributable to the abscopal effect (59). In HTM, the treatments did not significantly impaired the metastases formation. However, a clear tendency towards reduced lung metastasis was evident in JIMT-1 HTM when exposed to irradiation alone or in combination with atezolizumab. This implies that neoadjuvant tumor irradiation in HTM, and potentially in human patients, presumably does not enhance tumor cell migration, invasiveness, or dissemination. Instead, it appears to hinder distal tumor outgrowth. However, the duration of observation in this study is most likely insufficient to reliably assess the effect of irradiation on metastasis formation. Thus, additional long-term studies are required to investigate the ability of activated immune cells to prevent or eliminate systemic disease, specifically the dissemination and colonization of tumor cells at distant sides.

Finally, it is important to note that, associated with the greatest immune cell activation resulting from combining irradiation and anti-PD-L1 treatment, all HTM in this treatment group showed the most pronounced lack of weight gain or weight loss (**Supplementary Figure 3C**). Once more, this observation is in general consistent with the clinical situation (60). It can be assumed that immunomodulatory treatments not only cause anti-tumor effects but are also accompanied by immune cell activities that are not specific to the tumor, leading to side effects. Fortunately, humanized (tumor) mouse models have the ability to mirror both intended and unintended treatment effects (61). Accordingly, this mouse model helps to select the most effective and applicable therapeutic approaches before advancing to clinical translation (62).

Like other models, humanized tumor mice comprise limitation including the lack of MHC match between HSC and mouse tissue as well as to the tumor cells. However, also despite the full mismatch between the human leukocyte antigens (HLA) expressed on the HSCs and the mouse MHC expressed on tissues, the transplantation results in the generation of a human immune system without attacking the murine tissue. Even though T cells are generated in the mouse thymus and therefore are mostly H2-restricted, different authors reported human MHC dependent T cell activation in cord blood (CB) –reconstituted NOG (63), NSG (64) or RAG2-^/-^γc^-/-^ mice (65). In the context of MHC mismatched human immune system and tumor engraftment, Wang and colleagues reported that partially matched HSC transplantation allowed the engraftment of tumors and in most cases the growth kinetics of these tumors were not significantly different to immunodeficient controls, which were not engrafted with a human immune system (66). Wang also reported donor variation in response to anti-PD-1 therapy. Thus, it is very important to include different donors and splitting HTM generated by the same cord blood donor into all treatment groups. Different response rates to atezolizumab, which were also observed in our study, reflect the situation of responder and non-responder in the clinical setting. Accordingly, HTM are very helpful to identify biomarkers or predictive patterns associated with response or resistance. In addition, we overcame the non-responder situation by combination therapy with irradiation.

Multiple clinical trials were launched on the combination of checkpoint inhibitors and fractionated radiotherapy intending to enhance both a local and a systemic anti-tumor immune response. Such clinical trials were performed to evaluate treatments for non-small cell lung cancer (67), for head and neck squamous cell carcinoma (68), and for breast cancer (69, 70). Overall, the studies delivered promising data as the treatment was reported to be safe, well-tolerated and convincing response rates were seen. However, this combination therapy has not been implemented in standard treatment regimens yet.

In summary, our comprehensive treatment studies with HTM provide profound and valid evidence that neoadjuvant irradiation enhances the efficacy of anti-PD-L1 therapy of HER2-pos., triple negative, and ER-pos. BC cells. TNBC and HER2-pos. mice, initially not responding to checkpoint therapy can apparently be transformed into responders. An improved checkpoint therapy is achieved by an increased immunogenicity and immunity, coupled with diminished immunosuppression, both at the local and, most likely, at distant sites. In conclusion, we provide a solid foundation for translating the combined approach of irradiation and immunotherapy into the clinical setting as a feasible alternative to systemic (cytotoxic) treatment. Additional irradiation during immunotherapy has the potential to substantially contribute to the de-escalation of treatment.

## Data availability statement

The original contributions presented in the study are included in the article and in the **Supplementary Material**. Further inquiries can be directed to the corresponding author.

## Ethics statement

The studies involving humans were approved by Ethics Committee of the University of Regensburg. The studies were conducted in accordance with the local legislation and institutional requirements. The participants provided their written informed consent to participate in this study. The animal study was approved by District government of Bavaria. The study was conducted in accordance with the local legislation and institutional requirements.

## Author contributions

CB: Formal analysis, Methodology, Writing – original draft. VA: Methodology, Writing – original draft. SS: Resources, Writing – review & editing. SB: Formal analysis, Methodology, Writing – review & editing. KK: Formal analysis, Methodology, Writing – review & editing. FP: Methodology, Writing – review & editing. OO: Resources, Writing – review & editing. GB: Conceptualization, Funding acquisition, Methodology, Writing – original draft. AW: Conceptualization, Funding acquisition, Methodology, Writing – original draft.
