# Supplementary material for: Neoadjuvant radiotherapy in ER+, HER2+, and triple-negative -specific breast cancer based humanized tumor mice enhances anti-PD-L1 treatment efficacy
Source: Front Immunol. 2024 Apr 29;15:1355130. doi: 10.3389/fimmu.2024.1355130 (PMC11089195; doi:10.3389/fimmu.2024.1355130)
Supplement: Supplementary file 1 [file DataSheet_1.docx]

Supplementary Material
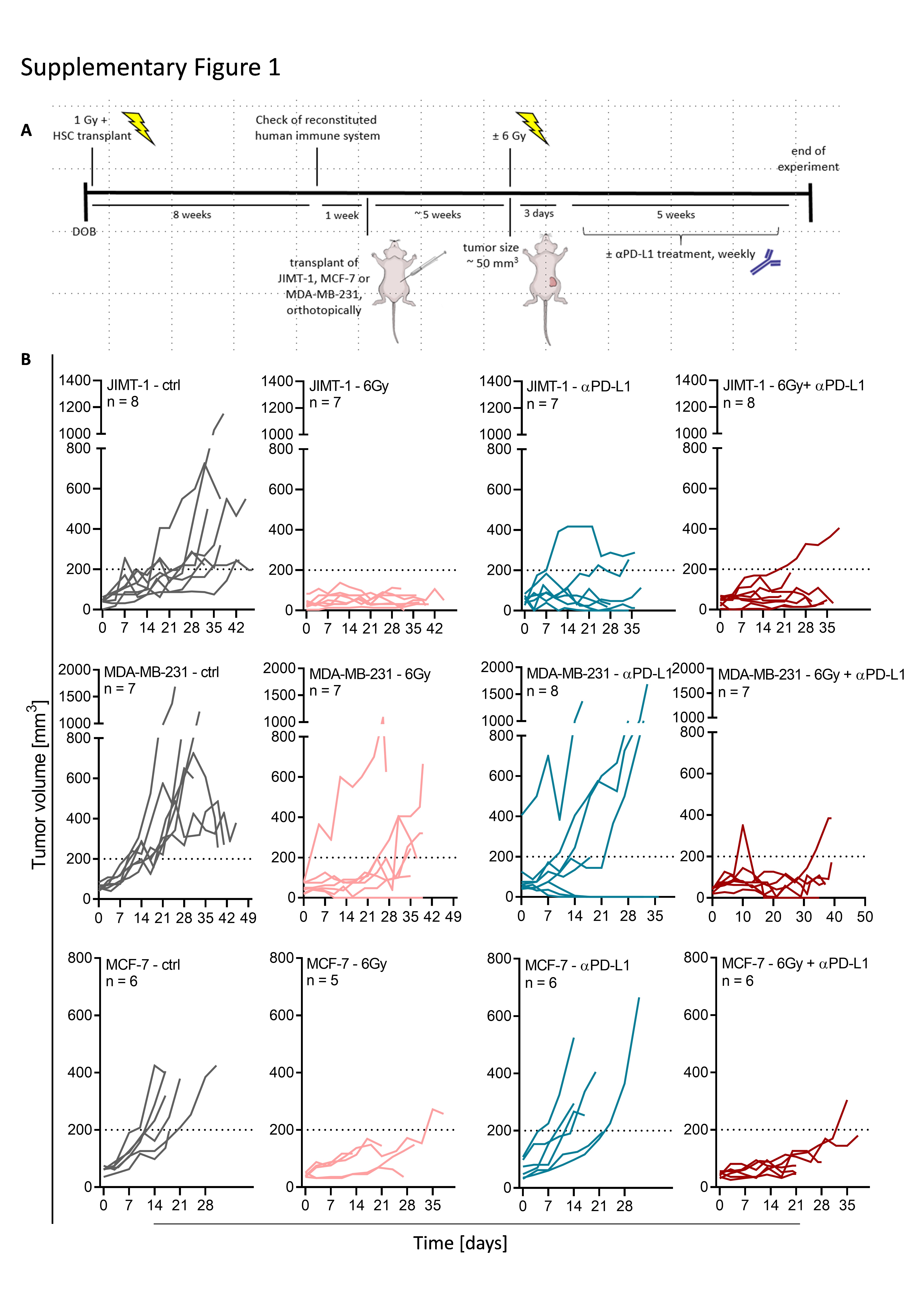


**Figure S1: Neoadjuvant irradiation alone reduced tumor growth, but subsequent checkpoint therapy augments in HTMs. (A)** Treatment regimen is depicted. Newborn NSG pups are irradiated with 1 Gy, and 1 × 10^5^ CD34^+^ cells are injected intra-hepatically. After eight weeks, blood is collected via the lateral saphenous vein and the state of the reconstituted human immune system is controlled. One week later, the mice are orthotopically transplanted with JIMT-1, MDA-MB-231, or MCF-7 breast cancer cells. Therapy is started when tumors are palpable (~ 5 mm in diameter). In case of irradiation, only the tumor areal is irradiated with 6 Gy. Anti-PD-L1 antibody (5 mg/kg body weight) is administered i. p. weekly, for five weeks. In the 6 Gy + anti-PD-L1 group, anti-PD-L1 administration is started three days after irradiation. **(B)** Tumor size was monitored over time, twice a week, n is given in the blots.


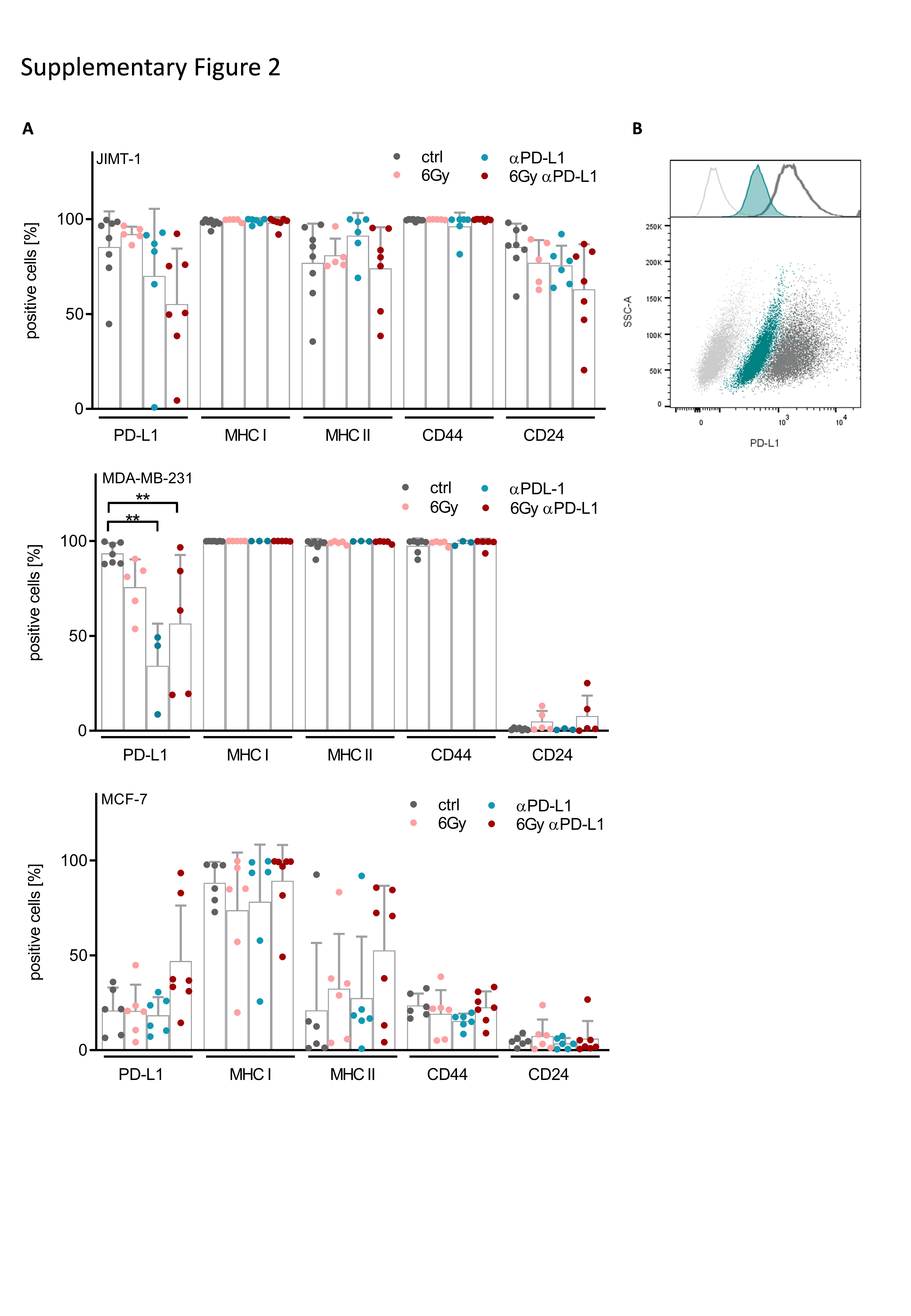


**Figure S2: Characterization of surface markers associated with the immune cell response and metastatic potential in tumors of HTMs. (A)** Treatment regimen see Figure 3 and S1. The tumors were harvested and processed to a single-cell suspension. Cells were subsequently analyzed by flow cytometry via EpCAM (JIMT-1, MCF-7) or ICAM (MDA-MB-231) and PD-L1, MHC I and II, CD24 and CD44 staining. One-way ANOVA, Tukey’s multiple comparisons test was applied, ** p ≤ 0.01. Data are shown as mean ± SD , each symbol represents an individual mouse under indicated conditions. **(B)** It is important to note, that competitive binding between atezolizumab and the diagnostic antibody may occur, as mice were sacrificed shortly after the last injection as shown with PD-L1 staining in MDA-MB-231 in vitro. Cells were incubated with or without atezolizumab (10 µg/ml) for 30 minutes, stained with anti-PD-L1 and subsequently analyzed by flow cytometry. An unstained control is shown in light grey. Cells incubated in the presence of atezolizumab are shown in blue, and in absence in dark grey (“ctrl”).


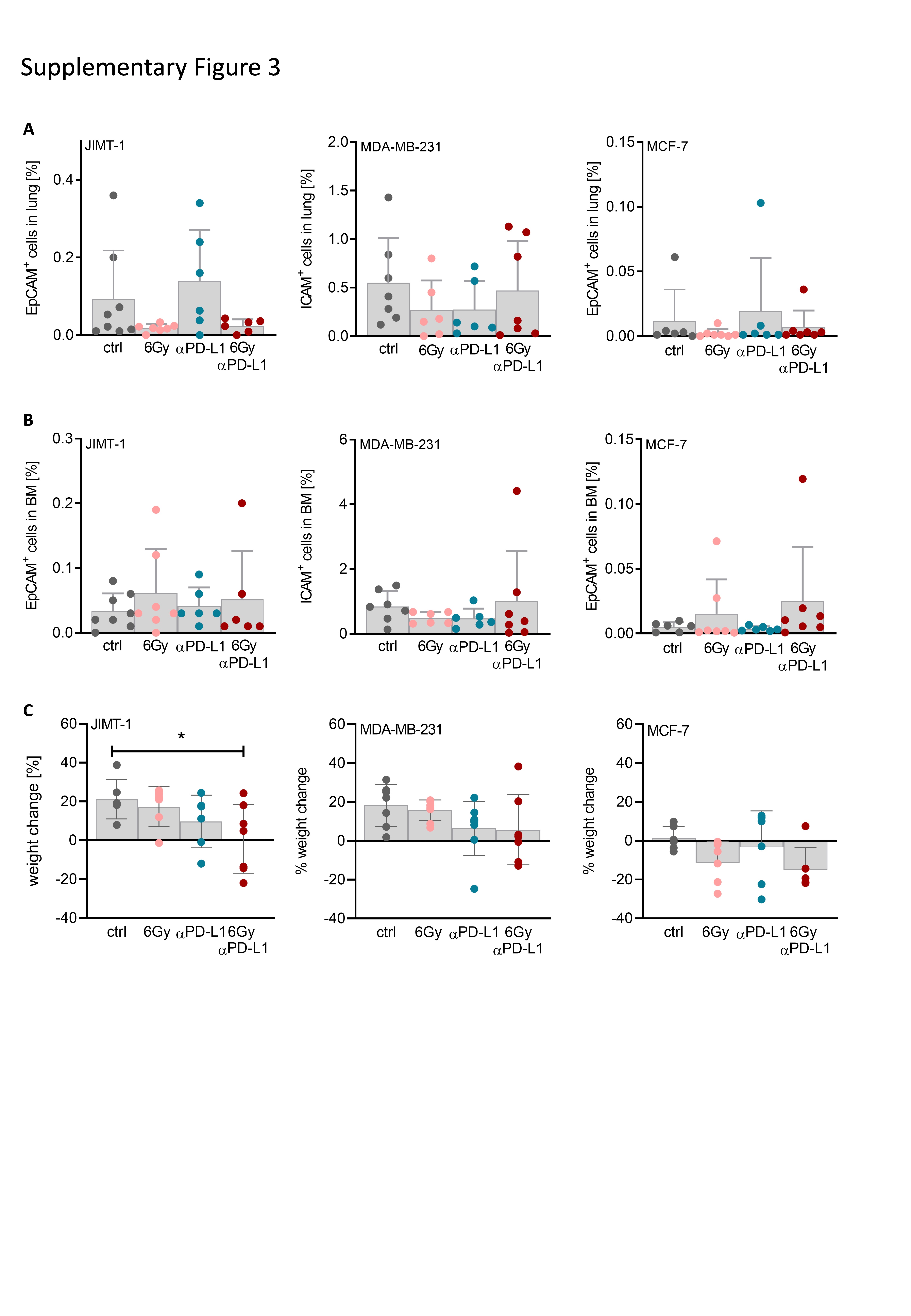


**Figure S3: Metastasis formation in lung and disseminated tumor cells in the bone marrow of HTMs with breast cancer.** Treatment regimen see Suppl. Fig. 1. **(A)** The lungs were harvested and processed to a single-cell suspension. **(B)** Bone marrow (BM) cells were isolated from the femur. Cells were subsequently analyzed by flow cytometry via EpCAM (JIMT-1, MCF-7) or ICAM (MDA-MB-231) staining. One-way ANOVA, Tukey’s multiple comparisons test was applied and did not show any significance. **(C)** Weight of the animals were monitored weekly and weight change starting from the time point of tumor transplantation until the end of the experiments are summarized as % weight change. Significances were analyzed using Dunnett’s multiple comparison test. Data are shown as mean ± SD, each symbol represents an individual mouse under indicated conditions. Data are shown as median, and differences to the ctrl group are depicted, * *p* ≤ 0.05.


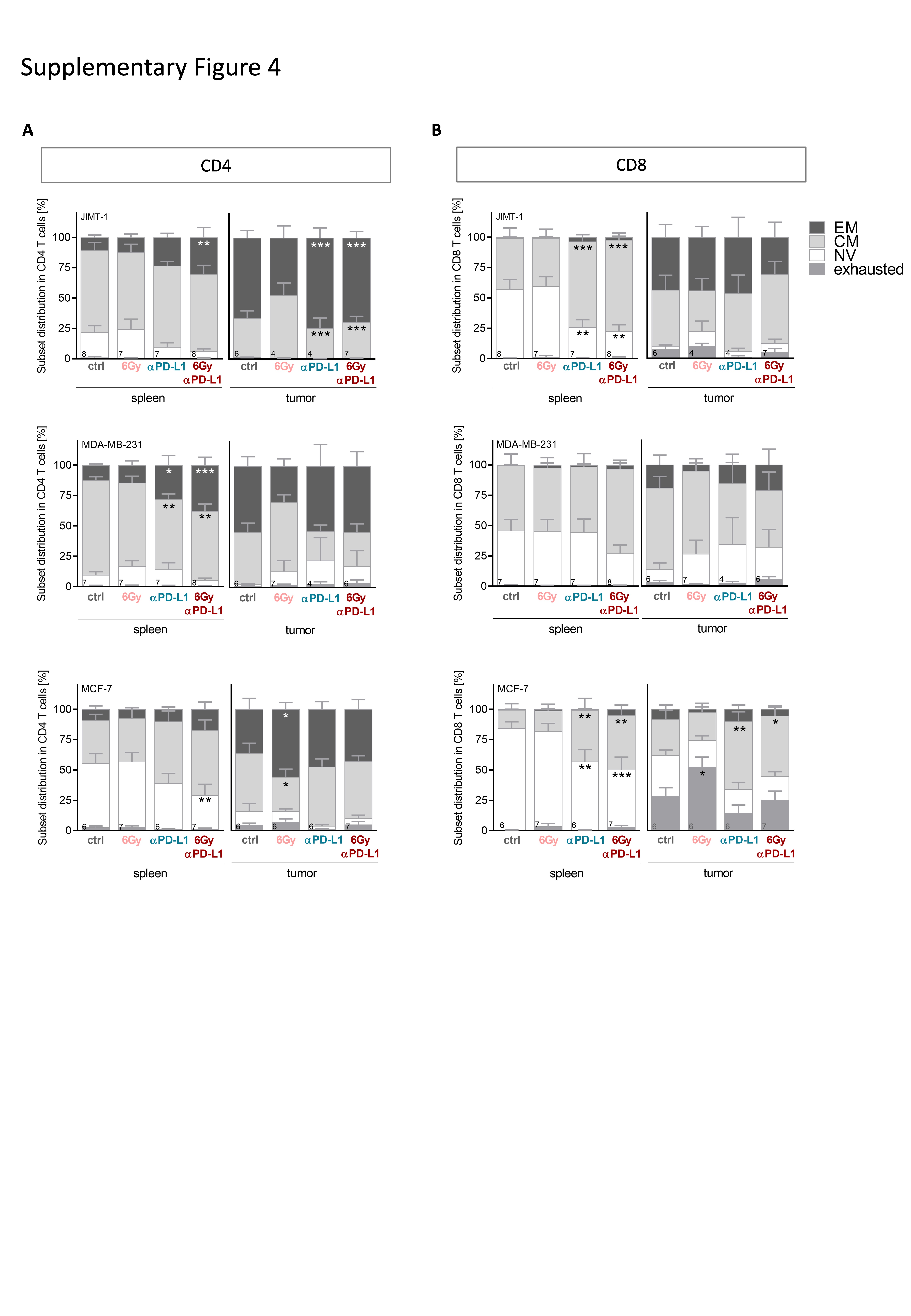


**Figure S4: Subset composition in the CD4 and CD8 T cell compartment changes after neoadjuvant irradiation and anti-PD-L1 therapy in HTMs with breast cancer.** Treatment regimen see Fig. 3 and Suppl. Fig. 1. The tumors and spleens were processed to a single-cell suspension and the cells were subsequently analyzed by flow cytometry by staining of CD4, CD8, CD27 and CD45RA, exhausted: CD27^–^ CD45RA^+^; naïve (NV): CD27^+^ CD45RA^+^; central memory (CM): CD27^+^ CD45RA^–^; effector memory (EM): CD27^–^ CD45RA^–^. **(A, B)** Subset distribution in the CD4 and CD8 T cell compartment, isolated from spleen and tumor under indicated conditions. Data are shown as mean ± SD, n is given in the lower left of each bar and two-way ANOVA, Tukey’s multiple comparisons test was applied, * *p* ≤ 0.05, ** *p* ≤ 0.01, *** *p* ≤ 0.001.

#
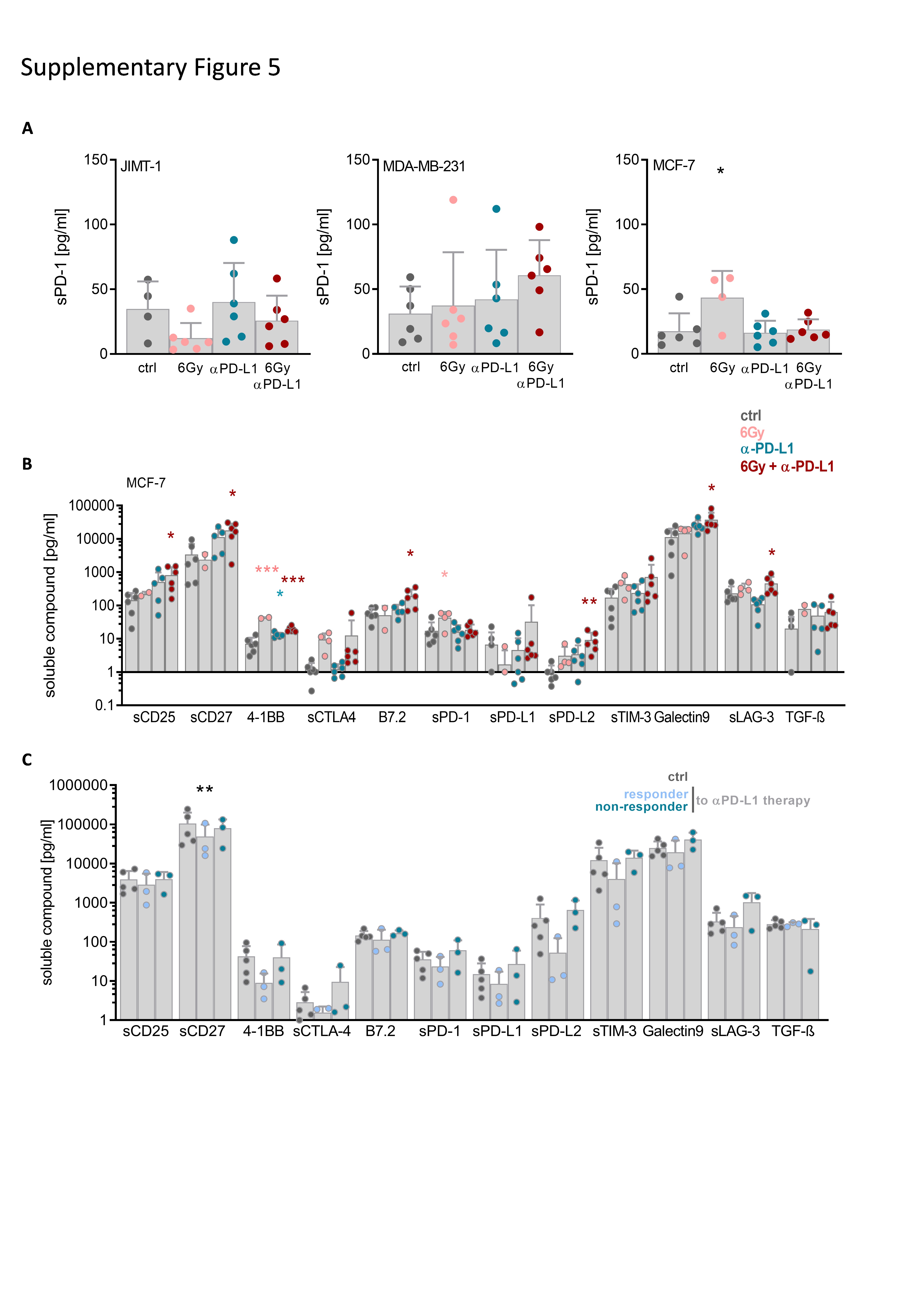


**Figure S5: Levels of soluble checkpoint molecules in serum of treated and untreated HTMs.** JIMT-1, MDA-MB-231 or MCF-7 breast cancer cells were transplanted orthotopically into humanized NSG mice. Treatment regimen see Suppl. Fig. 1. Five weeks after therapy was started, mice were sacrificed and serum was collected. Soluble factors in the murine serum of different proteins involved in the immune response were analyzed via bead-based immunoassays using flow cytometry, including human sCD25, sCD27, 4-1BB, CTLA-4, B7.2, sPD-1, sPD-L1, sPD-L2, sTIM-3, galectin-9, sLAG-3, and TGF-ß. **(A)** sPD-1 levels found in serum of the three HTM models were compared via one-way ANOVA, Tukey’s multiple comparisons test. **(B)** Levels found in MCF-7 HTMs were compared via one-way ANOVA, Tukey’s multiple comparisons test. **(C)** Levels found in MDA-MB-231 HTMs from the ctrl group as well as the responders and non-responders in the anti-PD-L1 were compared via two-way ANOVA, Šidák multiple comparisons test. **(A, B, C)** Data are shown as mean ± SD, and differences to the ctrl group are depicted, * *p* ≤ 0.05, ** *p* ≤ 0.01, *** *p* < 0.001.
